# An overview of sex hormones in relation to SARS-CoV-2 infection

**DOI:** 10.2217/fvl-2021-0058

**Published:** 2021-07-20

**Authors:** Marzieh Saei Ghare Naz, Mojdeh Banaei, Sareh Dashti, Fahimeh Ramezani Tehrani

**Affiliations:** 1 ^1^Reproductive Endocrinology Research Center, Research Institute for Endocrine Sciences, Shahid Beheshti University of Medical Sciences, Tehran, 1985717413, Iran; 2 ^2^Mother & Child Welfare Research Center, Hormozgan University of Medical Sciences, Bandar Abbas, 7916613885, Iran; 3 ^3^Department of Midwifery, Mashhad Branch, Islamic Azad University, Mashhad, 9187147578, Iran

**Keywords:** androgens, COVID-19, estradiol, female, genes, gonad, male, risk factor, sex specific, sex hormone

## Abstract

**Aim:** Sex differences in COVID-19 outcomes might be explained from a sex hormones (SexHs) perspective. **Materials & methods:** PubMed, Scopus, Web of Science, EMBASE and Google Scholar were searched up to March 2021. **Results:** Based on the literature review, the crosstalk between SexHs (estrogens, progesterone and testosterone), their receptors (estrogen α and β, androgen, and progesterone) and the immune system shaped the sex-related differences in immune responses against COVID-19. Differential production of SexHs over the lifespan (during pregnancy, reproductive years, menopause and andropause) and over different seasons may result in disparities in body response toward COVID-19. Moreover, SexHs-specific differences might affect vaccine efficacy and response to treatment. **Conclusion:** The roles of SexHs need to be considered in vaccine development and even treatment of COVID-19.

## Lay abstract

Female and male sex hormones (SexHs) play a pivotal role in the body’s defense against viral infections. SexHs and their binding molecules are among the main possible indicators of sex disparities in COVID-19 severity and response to treatment. Changes in the levels of SexHs from the childbearing age to menopause and pregnancy in females, and the aging process in males affect the immune function. This article sheds light on how physiologic hormonal differences in different sexes and in different stages of life affect COVID-19 severity. As a consequence the role of SexHs should be considered in vaccine development and treatment of COVID-19, especially for patients who suffer from hormone-related medical conditions in different stages of life.

SARS-CoV-2 is an ongoing health problem. The sex distribution of COVID-19 is unequal, and worse outcomes have been reported in men [1–6]. Similar sex differences are observed in other viral infections [7]. Various rationales, including biological differences in the immune system and behavioral factors, have been proposed to explain sex differences in COVID-19 morbidity and mortality [8]. Sex differences in COVID-19 severity and complications might also be explained from sex hormones (SexHs) perspective. Furthermore, the link between SexHs and respiratory infections is well established [9].

Sex stratification is one of the main parameters in disease development, progression and treatment [10,11]. Note that hormonal factors are among the fundamentally important biological determinants of health [12]. Gonadal hormones may act as a disease trigger and modulator [13], and also affect the host immune function [14]. Evidence suggests that androgen receptor (AR), angiotensin-converting enzyme 2 (ACE2) and transmembrane serine protease 2 contribute to the pathophysiology of COVID-19 [15,16]. Furthermore, SexHs can regulate both ACE2 and transmembrane serine protease 2 [17]. Besides environmental and genetic factors, SexHs play a detrimental role in the complex mechanism of the body’s immune response [18].

It seems that SexHs are among the main factors related to the sex-specific response to COVID-19 [19–21]. To address SexHs in relation to SARS-CoV-2 infection, we have collected and discussed the findings of available literature. A clear understanding of the role of SexHs in COVID-19 development and progression can help design future observational and clinical studies to prevent and treat this disease.

## Materials & methods

A narrative review was undertaken in accordance with the scale for the quality assessment of narrative review articles [22]. Electronic databases including PubMed, Scopus, Web of Science, EMBASE, Cochrane Library and Google Scholar were searched for relevant studies up to March 2021.

The search was conducted using the following key terms: ‘gonadal steroid hormones’, ‘hormones, gonadal steroid’, ‘steroid hormones, gonadal’, ‘sex steroid hormones’, ‘hormones, sex steroid’, ‘steroid hormones, sex’, ‘sex hormones’, ‘testosterone’, ‘progesterone’, ‘estradiol’, ‘androgens’, ‘gonad’, ‘ovary’, ‘testis’, ‘severe acute respiratory syndrome coronavirus 2’, ‘2019-ncov’, ‘Wuhan coronavirus’, ‘SARS-CoV-2’, ‘2019 novel coronavirus’, ‘COVID-19 virus’, ‘coronavirus disease 2019 virus’, ‘COVID19 virus’, ‘Wuhan seafood market pneumonia virus’ and ‘2019-nCoV’.

The search was limited to articles written in English. Researchers also performed a manual search in the references of the retrieved articles.

The titles and abstracts of the studies were reviewed for eligibility. The eligibility criteria included studies that investigated the relationship between SexHs and COVID-19. Observational and experimental studies, opinions, commentaries and letters to the editor, review papers, and electronic books which investigated whether SexHs affect the host’s immunity, COVID-19-related gene expression and COVID-19 infection were included in this review.

## Results & discussion

The results are presented and discussed in four sections: sex-specific production of SexHs, sex-specific immunoregulatory effects of SexHs, chromosome-linked genes and sex-specific production of SexHs, and SexHs and pre-existing chronic diseases related to hormone disorders in the context of COVID-19.

### Sex-specific production of SexHs

Human and animal studies have provided evidence that the levels of SexHs may have multiple effects on lung development and function, the host’s resistance to different parasites, susceptibility to respiratory infections and numerous lung diseases [23–25]. Note that the levels of SexHs can modulate the host’s immune response and respiratory control measures [26,27]. In general, similar SexHs including estrogens, progesterone and testosterone, are produced in male and female bodies, but these hormones have different serum levels, physiological functions, and target tissues in males and females [28,29]. In males, testosterone is mainly produced in testes with the diurnal rhythm; also, a slight amount of estrogens and progesterone is produced in the male body [30–32]. In contrast, in females, estrogens and progesterone are mainly produced in the ovaries in a cyclical pattern, while a slight amount of testosterone is produced by the ovaries and adrenal glands [28,29,33]. Through the developmental process, minimal presentation of SexHs has been shown during infancy; however, with the onset of adolescence, the level of SexHs rises during the reproductive aging, and the cyclic rise and fall of SexHs end by a transition to menopause phase (minimal presentation of estradiol and progesterone) [34]. Indeed, increased levels of SexHs are observed in the critical period of pregnancy [34]. SexHs also contribute to age-specific immune system response; therefore, menopause, andropause and SexH-related changes alter the immune activity [35].

It is evident in a study that, males are more susceptible to respiratory infections in all age groups (childhood and adulthood) than females [23]. This possible variation is explained by variations in the levels of SexHs, anatomic and lifestyle factors [23]. High testosterone levels due to facilitating viral entry could serve a potentially unfavorable role against COVID-19 in males. Surprisingly, the low testosterone levels in males also play an unfavorable role against COVID-19 [36]. In contrast, in females, progesterone and estrogens might show antiviral activity [36]. High 17β-estradiol (E2) and progesterone (P4) levels might improve immune response against COVID-19 [37]. Therefore, SexHs levels may serve as a double-edged sword against COVID-19.

To date, limited evidence exists regarding the effect of COVID-19 on testosterone levels. However, based on the existing evidence, androgen levels might affect ACE2 expression [38,39]. A recent study in Turkey on 44 patients (24 positive and 20 negative for COVID-19) demonstrated that the total testosterone (TT) levels decreased in patients with COVID-19 pneumonia [40]. A study conducted in 2010 demonstrated that TT was decreased in males with respiratory tract infections [41]. Furthermore, there is evidence that in patients with respiratory tract infections without COVID-19, TT levels were higher than in patients with COVID-19 infection, but both groups had lower levels of TT compared with the control group [42]. A recent cohort study by Dhindsa *et al.* on 90 males and 62 females with COVID-19 revealed that lower testosterone levels in males were associated with increased severity of COVID-19, inflammatory markers and disease-related mortality [43]. Another study also demonstrated that low TT and calculated free testosterone at admission were associated with poor outcomes [44]. These findings could be explained by the possible role of testosterone in inducing ACE2 as a lung-protective enzyme [45], and testosterone’s anti-inflammatory role through increasing cytokines [46,47]. However, the exact underlying mechanism is unknown. Overall, testosterone levels may affect COVID-19 progression and severity through its potential role in the regulation of the immune system or its effect on viral entry and fusion [48].

There is no clear evidence regarding the regeneration of lung tissue after the clearance of COVID-19 infection. Moreover, the role of androgens in repairing and generating lung tissue after damage due to the infection is unclear. It is evident in a study that innate immune cells greatly contribute to repairing the virus-induced inflammation [49], while the androgen and AR play a significant role in the innate and adaptive immune system [50]. In addition, it is well known that androgen is critical in delaying lung maturation during fetal life, by acting on lung fibroblast cells [51]. The exact role of androgens in adult human lung function is not fully understood. Evidence from unrelated studies showed that anti-androgenic therapy by spironolactone was effective in males affected by COVID-19. Importantly, the potential mechanism of anti-androgen treatments, including spironolactone, is accomplished through the antihyperinflammatory and antifibrotic effects of these treatments on fibroblasts [52]. On the other hand, there is evidence that ARs exist on the lung fibroblasts [53]. Further studies are required to identify the exact role of androgens on lung fibroblasts.

Numerous studies showed that seasonal variations in the level of SexHs may be observed in both males and females [54,55]. Andersson *et al.* found that testosterone levels were at the lowest level in males from winter to early spring [55]. Moreover, Bjørnerem *et al.* found 0.2–0.9% seasonal variation in estradiol in circulation in both males and females which peaked in June and May in postmenopausal women and men, respectively, while the nadir was observed in October in both sexes [54]. It is hypothesized that seasonal change in the levels of SexHs may affect the COVID-19 pandemic in terms of prevention and treatment. Therefore, it is recommended that future studies investigate the role of this variation in COVID-19 vaccine response to improve COVID-19 control.

In terms of the variation in drug reactions, recent data showed that physiological rise and fall in the levels of SexHs throughout the lifespan (aging, puberty and pregnancy) might affect drug response [56]. Therefore, response to treatment in patients with COVID-19 might be affected by SexHs-related factors.

Recently, the effect of SexH therapy has been investigated in COVID-19, and there are ongoing clinical trials to examine the impact of SexHs on COVID-19. Studies have investigated and discussed the possible therapeutic role of spironolactone, an anti-androgenic agent, in COVID-19 [57,58]. Estrogens and progesterone are other candidates against COVID-19 [37]. Considering the important role of SexHs, clinicians and researchers should pay more attention to sex differences in the dose and scheduling of COVID-19 vaccination [59].

In summary, differences in the production of SexHs might lead to a disparity between females and males in terms of dealing with the infection. Note that sex-specific production of SexHs during different life stages might contribute to COVID-19 onset, progression, and even vaccine efficacy and drug response. The role of SexHs should be considered in the design of effective COVID-19 prevention strategies. Future trials are recommended to better understand the risk/benefit of SexHs-based vaccination.

### Sex-specific immunoregulatory effects of SexHs

SexHs may affect the immune system’s response against pathogens [60], and therefore, lead to sex-specific immunoregulatory effects, which can be due to variations in the function and number of B and T lymphocytes, monocytes, granulocyte, natural killer cells, and the production of TNF-α, IL-1β, IFN-γ, and IL-2, IL-4, IL-6, IL-10, IL-12 and IL-18 between sexes [61]. Immune responses vary in humans depending on hormonal variation across reproductive phases [61]. In other words, the levels of SexHs contribute to immune regulation and response in both innate and adaptive immunity [62].

SexH receptors (estrogen receptors α and β, AR, and progesterone receptor) may act as rate-limiting points in immune activity [63]. Testosterone and estrogen act as regulators in immune function [64]. Besides, progesterone and testosterone similarly suppress immunity, both through innate and cell-mediated immune responses [20]. Progesterone suppresses Th1 response and supports the production of Th2 cytokines [20]. Estrogen receptors induce powerful humoral and cellular immune responses [65,66]. Similarly, estrogen was found to contribute to upper and lower airways function [67–69]. Therefore, a cyclic variation has been observed for SexHs during the menstrual cycle, which is found to affect respiratory functions [69].

Female SexHs including estradiol and progesterone, act as a regulator in innate and adaptive immunity [70]. It seems that females can be more resistant to viruses. This finding may be because estrogens can stimulate the immune system by modulating the function of B cells and the production of Th1 pro-inflammatory cytokines and Th2 anti-inflammatory cytokines [61,71,72]. In patients with COVID-19, the virus induces the vascular process in the lungs, while estradiol plays a possible protective role by positively modulating vascular responses [73]. In the same vein, Suba discussed the protective immune response of estrogen against COVID-19 and highlighted its role in preventive and therapeutic approaches [74].

A female’s life stage, based on the reproductive cycle, begins with menstruation and ends with menopause. Based on the evidence, female’s reproductive cycle (menstruation, ovulation and pregnancy) affects the immune system [61]. It is well established that dramatic changes in the level of SexHs lead to immune response changes against viral respiratory infections [9]. Therefore, immune-cell count and activity may change during the menstrual cycle because of hormonal fluctuation [75]. Females in the progesterone-dominant phase of the menstrual cycle (mid-luteal phase) are more susceptible to infectious diseases [76]. A case report of two female COVID-19 patients illustrated that the positive results of the reverse transcriptase‐PCR (RT‐PCR) test during the first menstrual period turned negative after hospitalization in one case, and in another case, the negative RT-PCR test turned positive before hospitalization during the first menstrual period [77]. Furthermore, a cross-sectional study by Ding *et al.* in China found that estradiol (E2) had an inverse correlation with COVID-19 severity [78]. Moreover, recent reports highlight that pregnant women are more prone to COVID-19 infection because of dramatic changes in SexHs and increased expression of ACE2 during pregnancy [79,80]. Female menopause status also acts as a risk factor for COVID-19 [78]. Endogenous and exogenous estrogens may activate the innate and cell-mediated immune responses, but testosterone and progesterone suppress the immune system [81]. Lung immune cells including dendritic cells, macrophages, neutrophils and eosinophils, are also affected by the expression and function of SexH receptors [82]. Further studies are required to compare different treatment approaches by considering the immunoregulatory effects of SexHs.

There are relevant questions regarding the role of testosterone levels (low/high) in the COVID-19 pandemic [21]. Polycystic ovary syndrome is a common endocrine disorder in women of reproductive age, and is characterized by hyperandrogenism. Higher male SexHs in the polycystic ovary syndrome population may affect their susceptibility to COVID-19 and the severity of COVID-19 outcomes [83]. Recent reports from different countries established that males are more prone to adverse outcomes of COVID-19 [84], even at a similar prevalence of infection in both sexes [6]. Testosterone has a suppressive immune action [85]. It is suggested that vaccines and therapies targeted to increase T cell in males might benefit from an immune response against COVID-19 [86].

In summary, the physiological crosstalk between SexHs and immune cells contributes to releasing cytokines [87]. From a biological point of view, male and female SexHs over the life course also affect the immune response to vaccines [88]. Different immune responses in males and females may result in differences in vaccine effectiveness [89]. Therefore, researchers and clinicians should consider the importance of sex differences in COVID-19 vaccine development [59].

### Chromosome-linked genes & sex-specific production of SexHs

Both SexH-specific gene expression and SexH alterations contribute to the pathology and immune response to different disorders [90–92]. The X chromosome contains the largest number of immune-associated genes [93]; therefore, double X-chromosomes in females may lead to a more robust immune response. However, the ubiquitous expression of ACE2 may damage reproductive function in females with COVID-19 [94]. ACE2 is highly expressed in testes as an androgen secretion source [95,96], indicating that SARS-CoV-2 may cause testicular dysfunction [97,98]; this effect may disrupt the reproductive capacity in infected men.

ACE2 acts as a host receptor for COVID-19 infection, and ACE2 gene variants may alter the susceptibility to this infection [99]. ACE2 has a sex chromosome-independent activity because its gene is located on the X chromosome [100]. SARS-CoV-2 enters the lungs via the ACE-2 receptor. There is a sex difference in the expression of ACE2 in the lung. It seems that modulation of ACE2 by SexH modulators could potentially be a supportive therapy for COVID-19 patients [101].

Another aspect that needs to be highlighted is that males have the potential to have the homozygous ACE2 gene [102]. In line with the findings mentioned above, Manning and Fink found that a high-digit ratio 2D:4D, which demonstrates high prenatal estrogen or low prenatal testosterone, could act as a possible risk factor for the severity of COVID-19 in males in 41 nations [103]. The possible explanation was related to the inverse correlation between male 2D:4D and the expression of ACE2 [103].

In summary, SexH-specific gene expression could affect the vaccine-related immune response [88]. Researchers should consider the importance of SexHs fluctuation over the life span.

### SexHs & pre-existing chronic diseases related to hormone disorders in the context of COVID-19

SexHs imbalance may describe different chronic conditions [104–106], which act as risk factors for COVID-19 and its poor outcomes. In fact, the fluctuation in SexHs may lead to sex-related differences in susceptibility to diseases, obesity and other factors, which are identified as risk factors for COVID-19 infection. For example, sex differences in leptin, insulin and estrogen play a critical role in the regulation of body weight [107]. SexHs may affect platelet activity and reactivity [108]. The potential role of platelet in COVID-19 has also been reported [109]. Notably, the immunomodulatory effects of SexHs could affect metabolic health [110]. On the other hand, some risk factors including obesity, diabetes, hypertension and various other medical conditions, are associated with poor outcomes in COVID-19 patients [111,112].

In summary, pre-existing chronic diseases related to hormone disorders may affect the immune response against COVID-19. To achieve the best health outcomes in males and females with pre-existing hormone-related chronic conditions, information should be obtained regarding the best dose selection of vaccines and drugs.

## Conclusion

COVID-19, a complex viral disease, may present and progress in various forms in male and female bodies. Synthesis, regulation and function of SexHs in two hormonal conditions (physiological hormonal changes during the lifespan, hormonal disorders and developing pre-existing chronic diseases) are linked to immune responses to COVID-19. SexH fluctuations during the lifespan may be responsible for the differences in immune system function against COVID-19. SexH-specific disparities could affect vaccine-related immune response and drug responses in patients with COVID-19. There are many gaps in the knowledge of the exact pathways explaining the association between SexHs and COVID-19 infection. Data on this may improve treatment and prevention modalities and provision of sex-based recommendations with possibly better outcomes.

## Future perspective

Regarding the concept of multiple roles of SexHs in COVID-19, sex-specified vaccines may be developed in the future. We anticipate that the researchers pay considerable attention to SexH-based modalities in the management of the COVID-19 pandemic. Considering the key role of SexHs in COVID-19, they are suggested as a target for designing SexHs-based interventions.

**Executive summary**

**Sex hormones levels**

- As a biological factor, the level of sex hormones (SexHs) in males and females may modulate the level of host response.

**Immunoregulatory effects of SexHs**

- SexH receptors, as well as testosterone, estradiol and progesterone concentrations may contribute to the immunoregulatory response to COVID-19. A better understanding of this complex pathway offers opportunities to provide better treatment strategies for both males and females.

**Chromosome-linked genes and SexHs**

- Sex chromosomes affect SexHs production, and thus, affect angiotensin-converting enzyme 2 and may alter the susceptibility to this infection.

**Pre-existing disorders and SexHs**

- Pathological changes in SexHs concentrations may affect the immune response against COVID-19.

## References

[1] Onder G, Rezza G, Brusaferro S. Case-fatality rate and characteristics of patients dying in relation to COVID-19 in Italy. JAMA 323(18), 1775–1776 (2020). 32203977 10.1001/jama.2020.4683

[2] Meng Y, Wu P, Lu W Sex-specific clinical characteristics and prognosis of coronavirus disease-19 infection in Wuhan, China: a retrospective study of 168 severe patients. PLoS Pathog. 16(4), e1008520 (2020). 32343745 10.1371/journal.ppat.1008520 PMC7209966

[3] Shim E, Tariq A, Choi W, Lee Y, Chowell G. Transmission potential and severity of COVID-19 in South Korea. Int. J. Infect. Dis. 93, 339–344 (2020). 32198088 10.1016/j.ijid.2020.03.031 PMC7118661

[4] Nasiri MJ, Haddadi S, Tahvildari A COVID-19 clinical characteristics, and sex-specific risk of mortality: systematic review and meta-analysis. Front. Med. 7, 459 (2020). 10.3389/fmed.2020.00459 PMC7385184 32793620

[5] Gadi N, Wu SC, Spihlman AP, Moulton VR. What’s sex got to do with COVID-19? Gender-based differences in the host immune response to coronaviruses. Front. Immunol. 11, 2147 (2020). 32983176 10.3389/fimmu.2020.02147 PMC7485092

[6] Jin J-M, Bai P, He W Gender differences in patients with COVID-19: focus on severity and mortality. Front. Public Health 8, 152 (2020). 32411652 10.3389/fpubh.2020.00152 PMC7201103

[7] Pradhan A, Olsson P-E. Sex differences in severity and mortality from COVID-19: are males more vulnerable? Biol. Sex Differ. 11(1), 1–11 (2020). 32948238 10.1186/s13293-020-00330-7 PMC7498997

[8] Acheampong DO, Barffour IK, Boye A, Aninagyei E, Ocansey S, Morna MT. Male predisposition to severe COVID-19: review of evidence and potential therapeutic prospects. Biomed. Pharmacother. 131, 110748 (2020). 33152916 10.1016/j.biopha.2020.110748 PMC7480230

[9] Kadel S, Kovats S. Sex hormones regulate innate immune cells and promote sex differences in respiratory virus infection. Front. Immunol. 9, 1653 (2018). 30079065 10.3389/fimmu.2018.01653 PMC6062604

[10] Mauvais-Jarvis F, Bairey Merz N, Barnes PJ Sex and gender: modifiers of health, disease, and medicine. Lancet 396(10250), 565–582 (2020). 32828189 10.1016/S0140-6736(20)31561-0 PMC7440877

[11] Westergaard D, Moseley P, Sørup FKH, Baldi P, Brunak S. Population-wide analysis of differences in disease progression patterns in men and women. Nat. Commun. 10(1), 666 (2019). 30737381 10.1038/s41467-019-08475-9 PMC6368599

[12] Vlassoff C. Gender differences in determinants and consequences of health and illness. J. Health Popul. Nutr. 25(1), 47–61 (2007). 17615903 PMC3013263

[13] Verthelyi D. Sex hormones as immunomodulators in health and disease. Int. Immunopharmacol. 1(6), 983–993 (2001). 11407317 10.1016/s1567-5769(01)00044-3

[14] Klein SL. The effects of hormones on sex differences in infection: from genes to behavior. Neurosci. Biobehav. Rev. 24(6), 627–638 (2000). 10940438 10.1016/s0149-7634(00)00027-0

[15] Hoffmann M, Kleine-Weber H, Schroeder S SARS-CoV-2 cell entry depends on ACE2 and TMPRSS2 and is blocked by a clinically proven protease inhibitor. Cell 181(2), 271–280.e278 (2020). 32142651 10.1016/j.cell.2020.02.052 PMC7102627

[16] Mjaess G, Karam A, Aoun F, Albisinni S, Roumeguère T. COVID-19 and the male susceptibility: the role of ACE2, TMPRSS2 and the androgen receptor. Prog. Urol. 30(10), 484–487 (2020). 32620366 10.1016/j.purol.2020.05.007 PMC7242948

[17] Foresta C, Rocca MS, Di Nisio A. Gender susceptibility to COVID-19: a review of the putative role of sex hormones and X chromosome. J. Endocrinol. Invest. 6, 1–6 (2020). 10.1007/s40618-020-01383-6 PMC7492232 32936429

[18] Taneja V. Sex hormones determine immune response. Front. Immunol 9, 1931–1931 (2018). 30210492 10.3389/fimmu.2018.01931 PMC6119719

[19] Majdic G. Could sex/gender differences in ACE2 expression in the lungs contribute to the large gender disparity in the morbidity and mortality of patients infected with the SARS-CoV-2 virus? Front. Cell. Infect. Microbiol. 10, 327–327 (2020). 32582576 10.3389/fcimb.2020.00327 PMC7295901

[20] Grandi G, Facchinetti F, Bitzer J. The gendered impact of coronavirus disease (COVID-19): do estrogens play a role? Eur. J. Contracept. Reprod. Health Care 25(3), 233–234 (2020). 32469251 10.1080/13625187.2020.1766017

[21] Pozzilli P, Lenzi A. Commentary: testosterone, a key hormone in the context of COVID-19 pandemic. Metab. Clin. Exp. 108, 154252–154252 (2020). 32353355 10.1016/j.metabol.2020.154252 PMC7185012

[22] Baethge C, Goldbeck-Wood S, Mertens S. SANRA: a scale for the quality assessment of narrative review articles. Res. Integr. Peer Rev. 4(1), 1–7 (2019). 30962953 10.1186/s41073-019-0064-8 PMC6434870

[23] Falagas ME, Mourtzoukou EG, Vardakas KZ. Sex differences in the incidence and severity of respiratory tract infections. Respir. Med. 101(9), 1845–1863 (2007). 17544265 10.1016/j.rmed.2007.04.011

[24] Carey MA, Card JW, Voltz JW, Germolec DR, Korach KS, Zeldin DC. The impact of sex and sex hormones on lung physiology and disease: lessons from animal studies. Am. J. Physiol. Lung Cell. Mol. Physiol 293(2), L272–278 (2007). 17575008 10.1152/ajplung.00174.2007

[25] Townsend EA, Miller VM, Prakash YS. Sex differences and sex steroids in lung health and disease. Endocr. Rev. 33(1), 1–47 (2012). 22240244 10.1210/er.2010-0031 PMC3365843

[26] Klein SL. The effects of hormones on sex differences in infection: from genes to behavior. Neurosci. Biobehav. Rev. 24(6), 627–638 (2000). 10940438 10.1016/s0149-7634(00)00027-0

[27] Behan M, Wenninger JM. Sex steroidal hormones and respiratory control. Respir. Physiol. Neurobiol. 164(1–2), 213–221 (2008). 18599386 10.1016/j.resp.2008.06.006 PMC2642889

[28] Ostojic S. Steroids: From Physiology to Clinical Medicine. BoD–Books on Demand, IntechOpen (2012).

[29] Hammes SR, Levin ER. Impact of estrogens in males and androgens in females. J. Clin. Invest. 129(5), 1818–1826 (2019). 31042159 10.1172/JCI125755 PMC6486327

[30] Brambilla DJ, Matsumoto AM, Araujo AB, Mckinlay JB. The effect of diurnal variation on clinical measurement of serum testosterone and other sex hormone levels in men. J. Clin. Endocrinol. Metab. 94(3), 907–913 (2009). 19088162 10.1210/jc.2008-1902 PMC2681273

[31] O'Donnell L, Stanton P, De Kretser DM. Endocrinology of the male reproductive system and spermatogenesis. : Endotext [Internet]. Feingold KR, Anawalt B, Boyce Aet al. (). mDText.com, Inc., MA, USA (2017).

[32] Hess RA. Estrogen in the adult male reproductive tract: a review. Reprod. Biol. Endocrinol. 1(1), 52 (2003). 12904263 10.1186/1477-7827-1-52 PMC179885

[33] Burger HG. Androgen production in women. Fertil. Steril. 77, 3–5 (2002). 10.1016/s0015-0282(02)02985-0 12007895

[34] Venkatesh KK, Cu-Uvin S. Anatomic and hormonal changes in the female reproductive tract immune environment during the life cycle: implications for HIV/STI prevention research. Am. J. Reprod. Immunol. 71(6), 495–504 (2014). 24832617 10.1111/aji.12247

[35] Gubbels Bupp MR, Potluri T, Fink AL, Klein SL. The confluence of sex hormones and aging on immunity. Front. Immunol. 9, 1269–1269 (2018). 29915601 10.3389/fimmu.2018.01269 PMC5994698

[36] Cattrini C, Bersanelli M, Latocca MM, Conte B, Vallome G, Boccardo F. Sex hormones and hormone therapy during COVID-19 pandemic: implications for patients with cancer. Cancers 12(8), 2325 (2020). 10.3390/cancers12082325 PMC7464909 32824674

[37] Mauvais-Jarvis F, Klein SL, Levin ER. Estradiol, progesterone, immunomodulation, and COVID-19 outcomes. Endocrinology 161(9), bqaa127 (2020). 32730568 10.1210/endocr/bqaa127 PMC7438701

[38] Chen J, Jiang Q, Xia X Individual variation of the SARS-CoV-2 receptor ACE2 gene expression and regulation. Aging Cell 19(7), e13168 (2020). 10.1111/acel.13168 PMC7323071 32558150

[39] Deng Q, Ur Rasool R, Russell RM, Natesan R, Asangani IA. Targeting androgen regulation of TMPRSS2 and ACE2 as a therapeutic strategy to combat COVID-19. iScience 24(3), 102254 (2021). 33681723 10.1016/j.isci.2021.102254 PMC7919514

[40] Okçelik S. COVID-19 pneumonia causes lower testosterone levels. Andrologia 53(1), e13909 (2021). 33210743 10.1111/and.13909 PMC7744831

[41] Muehlenbein MP, Hirschtick JL, Bonner JZ, Swartz AM. Toward quantifying the usage costs of human immunity: altered metabolic rates and hormone levels during acute immune activation in men. Am. J. Hum. Biol. 22(4), 546–556 (2010). 20309883 10.1002/ajhb.21045

[42] Kadihasanoglu M, Aktas S, Yardimci E, Aral H, Kadioglu A. SARS-CoV-2 pneumonia affects male reproductive hormone levels: a prospective, cohort study. J. Sex. Med. 18(2), 256–264 (2021). 33468445 10.1016/j.jsxm.2020.11.007 PMC7691132

[43] Dhindsa S, Zhang N, Mcphaul MJ Association of circulating sex hormones with inflammation and disease severity in patients with COVID-19. JAMA Netw. Open 4(5), e2111398–e2111398 (2021). 34032853 10.1001/jamanetworkopen.2021.11398 PMC8150664

[44] Rastrelli G, Di Stasi V, Inglese F Low testosterone levels predict clinical adverse outcomes in SARS-CoV-2 pneumonia patients. Andrology 9(1), 88–98 (2021). 32436355 10.1111/andr.12821 PMC7280645

[45] Rowland SP, O'Brien Bergin E. Screening for low testosterone is needed for early identification and treatment of men at high risk of mortality from COVID-19. Crit. Care 24(1), 367 (2020). 32560707 10.1186/s13054-020-03086-z PMC7303930

[46] Sansone A, Mollaioli D, Ciocca G Addressing male sexual and reproductive health in the wake of COVID-19 outbreak. J. Endocrinol. Invest. 44(2), 223–231 (2020). 32661947 10.1007/s40618-020-01350-1 PMC7355084

[47] Bianchi VE. The anti-inflammatory effects of testosterone. J. Endocr. Soc. 3(1), 91–107 (2019). 30582096 10.1210/js.2018-00186 PMC6299269

[48] Auerbach JM, Khera M. Testosterone’s role in COVID-19. J. Sex. Med. 18(5), 843–848 (2021). 33903045 10.1016/j.jsxm.2021.03.004 PMC7972673

[49] Gorski SA, Hufford MM, Braciale TJ. Recent insights into pulmonary repair following virus-induced inflammation of the respiratory tract. Curr. Opin. Virol. 2(3), 233–241 (2012). 22608464 10.1016/j.coviro.2012.04.006 PMC3378727

[50] Lai J-J, Lai K-P, Zeng W, Chuang K-H, Altuwaijri S, Chang C. Androgen receptor influences on body defense system via modulation of innate and adaptive immune systems: lessons from conditional AR knockout mice. Am. J. Path. 181(5), 1504–1512 (2012). 22959669 10.1016/j.ajpath.2012.07.008 PMC3483803

[51] Provost PR, Blomquist CH, Drolet RE, Flamand N, Tremblay Y. Androgen inactivation in human lung fibroblasts: variations in levels of 17β-hydroxysteroid dehydrogenase type 2 and 5α-reductase activity compatible with androgen inactivation. J. Clin. Endocr. Metab. 87(8), 3883–3892 (2002). 12161528 10.1210/jcem.87.8.8764

[52] Kotfis K, Lechowicz K, Drożdżal S COVID-19-the potential beneficial therapeutic effects of spironolactone during SARS-CoV-2 infection. Pharmaceuticals (Basel) 14(1), 71 (2021). 33477294 10.3390/ph14010071 PMC7830835

[53] Sultan C, Migeon BR, Rothwell SW, Maes M, Zerhouni N, Migeon CJ. Androgen receptors and metabolism in cultured human fetal fibroblasts. Pediatr. Res. 14(1), 67–69 (1980). 7360523 10.1203/00006450-198001000-00016

[54] Bjørnerem A, Straume B, Oian P, Berntsen GK. Seasonal variation of estradiol, follicle stimulating hormone, and dehydroepiandrosterone sulfate in women and men. J. Clin. Endocrinol. Metab. 91(10), 3798–3802 (2006). 16835279 10.1210/jc.2006-0866

[55] Andersson AM, Carlsen E, Petersen JH, Skakkebaek NE. Variation in levels of serum inhibin B, testosterone, estradiol, luteinizing hormone, follicle-stimulating hormone, and sex hormone-binding globulin in monthly samples from healthy men during a 17-month period: possible effects of seasons. J. Clin. Endocrinol. Metab. 88(2), 932–937 (2003). 12574235 10.1210/jc.2002-020838

[56] Moyer AM, Matey ET, Miller VM. Individualized medicine: sex, hormones, genetics, and adverse drug reactions. Pharmacol. Res. Perspect. 7(6), e00541–e00541 (2019). 31844524 10.1002/prp2.541 PMC6897337

[57] Cadegiani FA, Wambier CG, Goren A. Spironolactone: an anti-androgenic and anti-hypertensive drug that may provide protection against the novel coronavirus (SARS-CoV-2) induced acute respiratory distress syndrome (ARDS) in COVID-19. Front. Med. 7, 453 (2020). 10.3389/fmed.2020.00453 PMC7399048 32850920

[58] Cadegiani FA, Goren A, Wambier CG. Spironolactone may provide protection from SARS-CoV-2: targeting androgens, angiotensin converting enzyme 2 (ACE2), and renin-angiotensin-aldosterone system (RAAS). Med. Hypotheses 143, 110112 (2020). 32721806 10.1016/j.mehy.2020.110112 PMC7363620

[59] Mccartney PR. Sex-based vaccine response in the context of COVID-19. J. Obstet. Gynecol. Neonatal Nurs. 49(5), 405–408 (2020). 10.1016/j.jogn.2020.08.001 PMC7405769 32800743

[60] Van Lunzen J, Altfeld M. Sex differences in infectious diseases-common but neglected. J. Infect. Dis. 209(Suppl. 3), S79–S80 (2014). 24966193 10.1093/infdis/jiu159

[61] Bouman A, Heineman MJ, Faas MM. Sex hormones and the immune response in humans. Hum. Reprod. Update 11(4), 411–423 (2005). 15817524 10.1093/humupd/dmi008

[62] Taneja V. Sex hormones determine immune response. Front. Immunol 9(1931), 1–5 (2018). 30210492 10.3389/fimmu.2018.01931 PMC6119719

[63] Kadel S, Kovats S. Sex hormones regulate innate immune cells and promote sex differences in respiratory virus infection. Front. Immunol. 9, 1653 (2018). 30079065 10.3389/fimmu.2018.01653 PMC6062604

[64] Bhatia A, Sekhon HK, Kaur G. Sex hormones and immune dimorphism. Sci. World. J. 2014, 159150 (2014). 10.1155/2014/159150 PMC4251360 25478584

[65] Kovats S. Estrogen receptors regulate innate immune cells and signaling pathways. Cell. Immunol. 294(2), 63–69 (2015). 25682174 10.1016/j.cellimm.2015.01.018 PMC4380804

[66] Bird MD, Karavitis J, Kovacs EJ. Sex differences and estrogen modulation of the cellular immune response after injury. Cell. Immunol. 252(1–2), 57–67 (2008). 18294625 10.1016/j.cellimm.2007.09.007 PMC2544631

[67] Di Stadio A, Della Volpe A, Ralli M, Ricci G. Gender differences in COVID-19 infection. The estrogen effect on upper and lower airways. Can it help to figure out a treatment? Eur. Rev. Med. Pharmacol. Sci. 24(10), 5195–5196 (2020). 32495849 10.26355/eurrev_202005_21298

[68] Raghavan D, Jain R. Increasing awareness of sex differences in airway diseases. Respirology 21(3), 449–459 (2016). 26677803 10.1111/resp.12702

[69] Lomauro A, Aliverti A. Sex differences in respiratory function. Breathe (Sheff) 14(2), 131–140 (2018). 29875832 10.1183/20734735.000318 PMC5980468

[70] Beagley KW, Gockel CM. Regulation of innate and adaptive immunity by the female sex hormones oestradiol and progesterone. FEMS Immunol. Med. Microbiol. 38(1), 13–22 (2003). 12900050 10.1016/S0928-8244(03)00202-5

[71] Grimaldi CM, Jeganathan V, Diamond B. Hormonal regulation of B cell development: 17 beta-estradiol impairs negative selection of high-affinity DNA-reactive B cells at more than one developmental checkpoint. J. Immunol. 176(5), 2703–2710 (2006). 16493025 10.4049/jimmunol.176.5.2703

[72] Salem ML. Estrogen, a double-edged sword: modulation of TH1- and TH2-mediated inflammations by differential regulation of TH1/TH2 cytokine production. Curr. Drug Targets Inflamm. Allergy 3(1), 97–104 (2004). 15032646 10.2174/1568010043483944

[73] Breithaupt-Faloppa AC, Correia CDJ, Prado CM, Stilhano RS, Ureshino RP, Moreira LFP. 17β-Estradiol, a potential ally to alleviate SARS-CoV-2 infection. Clinics 75, e1980 (2020). 32490931 10.6061/clinics/2020/e1980 PMC7233687

[74] Suba Z. Prevention and therapy of COVID-19 via exogenous estrogen treatment for both male and female patients. J. Pharm. Pharm. Sci. 23(1), 75–85 (2020). 32324533 10.18433/jpps31069

[75] Oertelt-Prigione S. Immunology and the menstrual cycle. Autoimmun. Rev. 11(6–7), A486–A492 (2012). 22155200 10.1016/j.autrev.2011.11.023

[76] Alvergne A, Högqvist Tabor V. Is female health cyclical? Evolutionary perspectives on menstruation. Trends Ecol. Evol. 33(6), 399–414 (2018). 29778270 10.1016/j.tree.2018.03.006

[77] Zheng H, Tan J, Ma K, Meng W. Changes in RT-PCR test results and symptoms during the menstrual cycle of female individuals infected with SARS-CoV-2: report of two cases. J. Med. Virol. 93(1), 541–545 (2021). 32639581 10.1002/jmv.26275 PMC7361390

[78] Ding T, Zhang J, Wang T Potential influence of menstrual status and sex hormones on female SARS-CoV-2 infection: a cross-sectional study from multicentre in Wuhan, China. Clin. Infect. Dis. 72(9), e240–e248 (2020). 10.1093/cid/ciaa1022 PMC7454316 32697835

[79] Zhao X, Jiang Y, Zhao Y Analysis of the susceptibility to COVID-19 in pregnancy and recommendations on potential drug screening. Eur. J. Clin. Microbiol. Infect. Dis. 39(7), 1209–1220 (2020). 32328850 10.1007/s10096-020-03897-6 PMC7178925

[80] Dashraath P, Wong JLJ, Lim MXK Coronavirus disease 2019 (COVID-19) pandemic and pregnancy. Am. J. Obstet. Gynecol. 222(6), 521–531 (2020). 32217113 10.1016/j.ajog.2020.03.021 PMC7270569

[81] Grandi G, Facchinetti F, Bitzer J. The gendered impact of coronavirus disease (COVID-19): do estrogens play a role? Eur. J. Contracept. Reprod. Health Care 25(3), 233–234 (2020). 32469251 10.1080/13625187.2020.1766017

[82] Fuentes N, Silveyra P. Endocrine regulation of lung disease and inflammation. Exp. Biol. Med. (Maywood). 243(17–18), 1313–1322 (2018). 30509139 10.1177/1535370218816653 PMC6348592

[83] Kyrou I, Karteris E, Robbins T, Chatha K, Drenos F, Randeva HS. Polycystic ovary syndrome (PCOS) and COVID-19: an overlooked female patient population at potentially higher risk during the COVID-19 pandemic. BMC Med. 18(1), 220 (2020). 32664957 10.1186/s12916-020-01697-5 PMC7360476

[84] Gebhard C, Regitz-Zagrosek V, Neuhauser HK, Morgan R, Klein SL. Impact of sex and gender on COVID-19 outcomes in Europe. Biol. Sex Differ. 11(1), 1–13 (2020). 32450906 10.1186/s13293-020-00304-9 PMC7247289

[85] Trigunaite A, Dimo J, Jørgensen TN. Suppressive effects of androgens on the immune system. Cell. Immunol. 294(2), 87–94 (2015). 25708485 10.1016/j.cellimm.2015.02.004

[86] Takahashi T, Ellingson MK, Wong P Sex differences in immune responses that underlie COVID-19 disease outcomes. Nature 588(7837), 315–320 (2020). 32846427 10.1038/s41586-020-2700-3 PMC7725931

[87] Bhatia A, Sekhon HK, Kaur G. Sex hormones and immune dimorphism. Sci. World. J. 2014, 159150 (2014). 10.1155/2014/159150 PMC4251360 25478584

[88] Flanagan KL, Fink AL, Plebanski M, Klein SL. Sex and gender differences in the outcomes of vaccination over the life course. Annu. Rev. Cell Dev. Biol. 33, 577–599 (2017). 28992436 10.1146/annurev-cellbio-100616-060718

[89] Chang W-H. A review of vaccine effects on women in light of the COVID-19 pandemic. Taiwan J. Obstet. Gynecol. 59(6), 812–820 (2020). 33218394 10.1016/j.tjog.2020.09.006 PMC7486065

[90] Kur P, Kolasa-Wołosiuk A. Sex hormone-dependent physiology and diseases of liver. Int. J. Environ. Res. Public Health 17(8), 2620 (2020). 10.3390/ijerph17082620 PMC7216036 32290381

[91] Avila M, Bansal A, Culberson J, Peiris AN. The role of sex hormones in multiple sclerosis. Eur. Neurol. 80(1–2), 93–99 (2018). 30343306 10.1159/000494262

[92] Aloisi AM. Why we still need to speak about sex differences and sex hormones in pain. Pain Ther. 6(2), 111–114 (2017). 29058253 10.1007/s40122-017-0084-3 PMC5693815

[93] Bianchi I, Lleo A, Gershwin ME, Invernizzi P. The X chromosome and immune associated genes. J. Autoimmun. 38(2–3), J187–192 (2012). 22178198 10.1016/j.jaut.2011.11.012

[94] Jing Y, Run-Qian L, Hao-Ran W Potential influence of COVID-19/ACE2 on the female reproductive system. Mol. Hum. Reprod. 26(6), 367–373 (2020). 32365180 10.1093/molehr/gaaa030 PMC7239105

[95] Verma S, Saksena S, Sadri-Ardekani H. ACE2 receptor expression in testes: implications in coronavirus disease 2019 pathogenesis. Biol. Reprod. 103(3), 449–451 (2020). 32427288 10.1093/biolre/ioaa080 PMC7314215

[96] Fan C, Lu W, Li K, Ding Y, Wang J. ACE2 expression in kidney and testis may cause kidney and testis infection in COVID-19 patients. Front. Med. 7(1045), 563893 (2021). 10.3389/fmed.2020.563893 PMC7838217 33521006

[97] Selvaraj K, Ravichandran S, Krishnan S, Radhakrishnan RK, Manickam N, Kandasamy M. Testicular atrophy and hypothalamic pathology in COVID-19: possibility of the incidence of male infertility and HPG axis abnormalities. Reprod. Sci. 7, 1–8 (2021). 10.1007/s43032-020-00441-x PMC7790483 33415647

[98] Shen Q, Xiao X, Aierken A The ACE2 expression in Sertoli cells and germ cells may cause male reproductive disorder after SARS-CoV-2 infection. J. Cell Mol. Med. 24(16), 9472–9477 (2020). 32594644 10.1111/jcmm.15541 PMC7361928

[99] Benetti E, Tita R, Spiga O ACE2 gene variants may underlie interindividual variability and susceptibility to COVID-19 in the Italian population. Eur. J. Hum. Genet. 28(11), 1602–1614 (2020). 32681121 10.1038/s41431-020-0691-z PMC7366459

[100] Liu J, Ji H, Zheng W Sex differences in renal angiotensin converting enzyme 2 (ACE2) activity are 17β-oestradiol-dependent and sex chromosome-independent. Biol. Sex Differ. 1(1), 6–6 (2010). 21208466 10.1186/2042-6410-1-6 PMC3010099

[101] Majdic G. Could sex/gender differences in ACE2 expression in the lungs contribute to the large gender disparity in the morbidity and mortality of patients infected with the SARS-CoV-2 virus? Front. Cell. Infect. Microbiol. 10(327), 327 (2020). 32582576 10.3389/fcimb.2020.00327 PMC7295901

[102] Gemmati D, Bramanti B, Serino ML, Secchiero P, Zauli G, Tisato V. COVID-19 and individual genetic susceptibility/receptivity: role of ACE1/ACE2 genes, immunity, inflammation and coagulation. Might the double X-chromosome in females be protective against SARS-CoV-2 compared to the single X-chromosome in males? Int. J. Mol. Sci. 21(10), 3474 (2020). 10.3390/ijms21103474 PMC7278991 32423094

[103] Manning JT, Fink B. Understanding COVID-19: digit ratio (2D:4D) and sex differences in national case fatality rates. Early Hum. Dev. 146, 105074–105074 (2020). 32419720 10.1016/j.earlhumdev.2020.105074 PMC7224643

[104] Dubey RK, Oparil S, Imthurn B, Jackson EK. Sex hormones and hypertension. Cardiovasc. Res. 53(3), 688–708 (2002). 11861040 10.1016/s0008-6363(01)00527-2

[105] Henderson BE, Feigelson HS. Hormonal carcinogenesis. Carcinogenesis 21(3), 427–433 (2000). 10688862 10.1093/carcin/21.3.427

[106] Bruns CM, Kemnitz JW. Sex hormones, insulin sensitivity, and diabetes mellitus. ILAR J. 45(2), 160–169 (2004). 15111735 10.1093/ilar.45.2.160

[107] Shi H, Clegg DJ. Sex differences in the regulation of body weight. Physiol. Behav. 97(2), 199–204 (2009). 19250944 10.1016/j.physbeh.2009.02.017 PMC4507503

[108] Karolczak K, Konieczna L, Kostka T Testosterone and dihydrotestosterone reduce platelet activation and reactivity in older men and women. Aging (Albany NY) 10(5), 902–929 (2018). 29723157 10.18632/aging.101438 PMC5990384

[109] Koupenova M. Potential role of platelets in COVID-19: implications for thrombosis. Res. Pract. Thromb. Haemost. 4(5), 737–740 (2020). 32685881 10.1002/rth2.12397 PMC7283793

[110] Rubinow KB. An intracrine view of sex steroids, immunity, and metabolic regulation. Mol. Metab. 15, 92–103 (2018). 29551633 10.1016/j.molmet.2018.03.001 PMC6066741

[111] Wolff D, Nee S, Hickey NS, Marschollek M. Risk factors for Covid-19 severity and fatality: a structured literature review. Infection 49(1), 15–28 (2021). 32860214 10.1007/s15010-020-01509-1 PMC7453858

[112] Williamson EJ, Walker AJ. Factors associated with COVID-19-related death using OpenSAFELY. Nature 584(7821), 430–436 (2020). 32640463 10.1038/s41586-020-2521-4 PMC7611074

